# Defining Information Security

**DOI:** 10.1007/s11948-017-9992-1

**Published:** 2017-11-15

**Authors:** Björn Lundgren, Niklas Möller

**Affiliations:** Division of Philosophy, Royal Institute of Technology (KTH), Brinellvägen 32, 100 44 Stockholm, Sweden

**Keywords:** Information security, Defining information security, CIA definition, Human aspects on information security, Ethical aspects on information security, Appropriate access

## Abstract

This article proposes a new definition of information security, the ‘Appropriate Access’ definition. Apart from providing the basic criteria for a definition—correct demarcation and meaning concerning the state of security—it also aims at being a definition suitable for any information security perspective. As such, it bridges the conceptual divide between so-called ‘soft issues’ of information security (those including, e.g., humans, organizations, culture, ethics, policies, and law) and more technical issues. Because of this it is also suitable for various analytical purposes, such as analysing possible security breaches, or for studying conflicting attitudes on security in an organization. The need for a new definition is demonstrated by pointing to a number of problems for the standard definition type of information security—the so-called CIA definition. Besides being too broad as well as too narrow, it cannot properly handle the soft issues of information security, nor recognize the contextual and normative nature of security.

## Introduction

What is information security? This is the question that this article will attempt to answer, by proposing, and arguing for, a definition of said subject. However, this question is potentially ambiguous, since ‘information security’ could refer to many things, e.g., the name of an academic subject, a practice, a goal, or a state of affairs. Thus, in order to answer the question, it must first be disambiguated. The authors believe that the most fundamental and interesting question to answer is when some information (system)^1^ is secure, since unless one has an understanding of this core notion, one does not know what the ideal would be that one is striving towards in the information security domain. The aim of this paper is thus to define this state.

Such a definition of security should fulfil at least three conditions. First, it should supply necessary and sufficient conditions for the state of security of any information (system). Second, these conditions should capture the meaning, or sense, of the concept (thus matching a suitable *understanding* of the term to be defined). These two conditions encapsulate what Anil Gupta calls “three grades of descriptive adequacy of a definition: extensional, intensional, and sense”.^2^ The first condition, i.e. necessary and sufficient conditions, ensures that the definition is adequate both extensionally (i.e. it has no actual counter-examples) and intensionally (i.e. it has no possible counter-examples), while the second condition focuses on the sense of the concept.^3^ Supposing a definition has captured the adequate sense of the concept there is also a third condition; that it should be helpful in an analysis of security breaches, incidents, and security related value conflicts. This third condition is especially important for increasing the value of the definition beyond pure demarcation issues (as will be clear from the examples in the “Further Arguments for the AA Definition” section, the proposed definition may be helpful in analysing, e.g., security/privacy conflicts).

Before presenting the definition, however, the need for a new definition will first be motivated by demonstrating the shortcomings of the main antagonist in this paper: the so-called ‘CIA definition’, the dominating definition of information security in the literature.^4^ The CIA definition is based on the following triad of properties:

- Confidentiality: “property that information is not made available or disclosed to unauthorized individuals, entities, or processes”.
- Integrity: “property of accuracy and completeness”.
- Availability: “property of being accessible and usable upon demand by an authorized entity”.^5^

To these base properties others are sometimes added.^6^ Variations of these characterisations can be found in ISO-standards, US federal law and standards, as well as other national standards, handbooks, and articles.^7^ While the exact formulations vary, the critique here put forward is valid across the variations. For this reason, the CIA properties will henceforth be characterized in accordance with the ISO definitions.

In what follows, the idea that information security can be defined by the CIA properties will be challenged. However, a potential worry that is worth considering already at the outset is that the CIA triad is not intended as a definition.^8^ For example, some theorists argue that CIA supplies *goals* for a secure system rather than serving as a definition supplying a demarcation between secure and insecure information (systems). The problem with this retreat position is twofold. First, since the CIA properties are not necessary, the CIA-triad cannot properly function as goals either.^9^ Secondly, the CIA triad is de facto utilized as a definition in many international standards, as well as in many US standards, and is the textbook characterisation in the security profession. Consequently, the need for a proper analysis of the CIA triad as a definition—and a way forward should it be found wanting—is much needed. This is the task of the present paper.^10^

Before substantially analysing the CIA definition, one immediate problem needs to be overcome: the problematic form of the definition found in standard texts. In order to properly discuss potential counter-examples, the CIA definition will be modified so that it clearly states necessary and sufficient conditions. The authors propose the following specification:

*The CIA definition of secure information*: some information I is secure if, and only if, all parts of I retain the properties of confidentiality, integrity, and availability.

Henceforth, when the ‘CIA definition’ is mentioned it refers to this specification. This is a definition of absolute security, which is taken to be the most fundamental property to define and hence the main concern of the current paper.^11^ In practice, however, it is relevant to determine not only if security of some information (system) is breached, but to what degree it is breached or the likelihood that it is secure. For this reason, the extent to which the criticism also encompasses gradable versions of the CIA definition will be discussed below.

This paper is structured as follows. In the next section, the CIA definition is critically evaluated and it is shown how it is susceptible to counter-examples. It will be argued that these problems are rooted in its lack of a proper relation to an analysis of security. As history has shown, security is not only about technical solutions, but also about human actions and interactions. According to Gurpreet Dhillon and James Backhouse, a majority of breaches depend on insiders,^12^ indicating that ‘soft issues’—involving human, organizational, cultural, ethical, policy, or juridical aspects—play an important role.^13^ A definition of information security must be properly applicable to such analyses. Based on these counter-examples and the need for a more fundamental analysis of security, “The Appropriate Access definition” section proposes a new definition of information security that is suitably applicable also to the softer side of information security, and treats a number of potential objections to our definition. In the following section additional arguments for the definition are treated, which includes examples that show that the definition useful beyond pure security-issues. In the final section, our conclusions are summarised.

## Counter-Examples to the CIA Definition

The first and perhaps most fundamental problem with the CIA definition is that it is both too broad and too narrow, i.e. it defines insecure states as secure and secure states as insecure. This section will start by addressing the latter problem, arguing that the CIA triad does not supply necessary properties for information security.

Consider *availability*, i.e. the “property of being accessible and usable upon demand by an authorized entity”.^14^ There are genuine examples of devices intended to provide security that conflicts directly with—and as such contradicts the necessity of—availability. Take, e.g., time-locks: the whole idea of time-locks is that they provide security by making its content *unavailable*. Another example is the global seed vault on Svalbard. In this vault seeds are stored in the case of major catastrophes. At this moment, or in the foreseeable future, mankind do not need access to the seeds. Thus, in a more general sense the only purpose of the vault is to make sure that the integrity of the seeds remains, and if the seeds are inaccessible for some period of time—making them *not usable upon demand*—this would not affect the state of security. But, according to the CIA definition, it would. Cases such as these show that availability cannot be considered a necessary property for information security.

Now, a proponent of the CIA definition might perhaps argue that in the case of the time-lock one has simply given priority to the protection of integrity and confidentiality.^15^ While this may be the case, it actually supports rather than undermines the ‘too narrow’ point. If using a time-lock prioritizes confidentiality and integrity over availability *without* compromising security, then availability simply isn’t necessary for all kinds of information (systems). Therefore, the conclusion to draw here is that the CIA definition simply isn’t properly attuned to the different needs that different information (systems) have. While this is in practice recognized by many security experts, the definition fails to recognize this.

The other two properties of the CIA definition arguably face similar problems. Take integrity; here, a counter-example could be an open artwork similar to fridge magnet poetry where anyone could change the content of the information, so that the artwork is made to be whatever people make of it. While the integrity of the information would not be protected when the content is changed, it seems correct to say that the security of the artwork is still protected, since there is no *need* whatsoever to protect its integrity—rather the point is to compromise the integrity (in the CIA-sense of the term) of the artwork.^16^

Perhaps it may be argued that the fridge magnet artwork *is* insecure, because it lacks integrity, but that insecurity in this case is unproblematic, since the artwork does not *need* to be secure.^17^ In effect, this amounts to insisting that the notion of integrity is analytically tied to security. But while the authors can appreciate that intuitions about individual cases may vary, on closer inspection there is little reason—beyond old habit—to hold on to this intuition. In order for the term ‘insecure’ to apply to a system or piece of information, there should surely be something *undesirable* about the state of the system or information. And here the lack of integrity does not constitute something undesirable in any way, but is on the contrary something positively *desirable*. Consequently, the term ‘insecure’ seems to have little grip in a case like this.

In the case of the final property, *confidentiality*, it is important to note that a counter-example against confidentiality along the same lines as the previous examples is not attainable. A system which has no confidential information satisfies the property of confidentiality simpliciter, since if there is no confidential information then everyone is authorized to access any information and the property is satisfied. The problem, however, is that it is not clear that a loss of confidentiality necessarily implies (that there is or has been) a security breach (and consequently a loss of security). The property of confidentiality requires that no information is disclosed to unauthorized individuals. But imagine a case in which a person who *should*^18^ be authorised, and perhaps soon will be so, is—for expedience—disclosed information for which she is currently not authorized. While it constitutes a security breach according to the CIA definition (and plausibly an action in conflict with the security policy of the organisation), it seems that security is not breached if this person *should* have been authorised. Just because a person is not (yet) authorized by an organization doesn’t mean that her access would be improper for that organization (just as a person that is authorized perhaps shouldn’t be, if one aims to retain the security of a certain organization, as the examples of Manning and Snowden have shown).

A recent security scandal also exemplifies a parallel case. The scandal concerned the outsourcing of the Swedish Transport Agency’s IT operations. Part of the problem was that “foreign personnel who didn’t have Swedish security clearance gaining access to classified information”.^19^ While this clearly violates the confidentiality criteria it is, however, not clear that the information has not been—otherwise—handled correctly. Indeed, imagine that the personnel would have passed a security clearance (if they had been properly vetted). Suppose it was true, then it seems counter-intuitive to claim that the information is insecure *only* because this was not done earlier. Thus, although this was a violation of Swedish law it is not obvious that the security has actually been breached.

There is a substantial discussion to be had on the role of a gradable conception of security in situations like these. However, if it is correct that the CIA properties are not necessary, then a gradable conception of security in terms of the CIA properties does not work either, since, e.g., if a property is not necessary making it less likely that it is retained does not necessarily lead to decreased security either.

More can be said about the properties of integrity and confidentiality, in particular—which admittedly require a more substantial discussion to be conclusive—but in order not to foreshadow the positive arguments for the *Appropriate Access* definition (treated in later sections), the issue will not be further pursued here. Turn instead from the ‘too narrow’ aspect of the criticism to the ‘too broad’ aspect: i.e. the claim that the CIA definition defines insecure states as secure. In other words, it entails *false positives*.

Take the case of Edward Snowden. While the details of how Snowden managed to gather all of these confidential documents are not widely known, some claim, for example, that Snowden fabricated a few digital keys, meaning that already at the gathering process he had affected the system integrity (and therefore the security according to the CIA definition).^20^ However, one can imagine a situation in which Snowden didn’t have to perform any hacks (digital or otherwise, i.e. by eliciting non-authorized access by other means) in order to gather the documents. In other words, he would then be authorized to access (and, therefore, gather) all the documents that he actually gathered. Let the person in this (plausible) counterfactual situation be known as ‘c-Snowden’.^21^ In accordance with the CIA definition it would not have been a security breach for c-Snowden to gather the documents (since he was authorized to access them all). Instead, according to the CIA definition, security was breached by c-Snowden only when he passed the secret documents to a non-authorized third-party (‘T’), since only then would the confidentiality of the information be breached.

It seems more plausible to say, however, that the security breach happened *before* c-Snowden actually shared the secret documents. Imagine two possible scenarios:

(S1): c-Snowden gives the secret documents to T.
(S2): c-Snowden intends to give documents to T, but on his way to do so, he ends up in a traffic accident and the plan is uncovered. As a result of this the information is retrieved before any non-authorized user could access it.

Now, according to the CIA definition it would be the *sharing* of the information that is the breach; consequently, security is breached in S1, but not in S2. But that seems counter-intuitive, since the intention and preparatory act alone should suffice for there to be a security breach in S2. If one asked those who retrieved the documents if there had been a security breach (or incident), answering ‘no’ hardly would seem proper. What they should say is that ‘yes, there was a security breach, but the documents are now secure’. It seems, though, that the CIA definition here gets the cart before the horse: it is the breach of security that enables the sharing of confidential information, not the other way around. While a breach enabled sharing in both S1 and S2, circumstances disabled sharing in S2. Thus, that confidentiality wasn’t retained (in S1) is a consequence of a security breach, not the security breach itself. In the case of S2 what one should say is that the security was breached, but that the breach had non-severe consequences, since the confidentiality of all the documents were retained (and the documents are now secure).

In order to save the CIA definition, one might argue that the above examples only work if one considers an absolute sense of security, or one might try to say that the serious consequences of S1 is what makes it a security breach and that the lack of serious consequences of S2 explains why it isn’t a security breach. The idea then would be that the CIA-triad defines such serious consequences. However, just as one should not conflate the consequences of a security breach with what constitutes the breach itself, one should not conflate the normative evaluation of the consequences of a security breach with what constitutes the breach itself. Nevertheless here follows yet another argument against this possibility that also cuts against the idea that the CIA definition can be used to define a gradable conception of security, e.g., according to which some information (system) is *secure to the degree* that it is likely to retain the CIA properties. Consider the following situation:

(S3): c-Snowden gives the secret documents to T, but before T can do anything with the documents the incident is uncovered, and during a police raid T is shot dead, and as result of this the information is retrieved before any further non-authorized user could access it.

In S3, confidentiality is breached and thus according to the CIA definition security is breached. However, the results of the incident are only as serious as (or less than, since T is eliminated and that threat is therefore gone) that of S2, so it would be a mistake to think that focusing on consequences might save the CIA definition. This also illustrates why a gradable version of the CIA definition does not help, since in S3 confidentiality is breached, meaning that the likelihood of it being breached is 100%. Yet the information is not less secure in S3 than in S2 and S1.^22^

Returning to the issue of mistakenly conflating the *consequences* of a breach with the actual *breach* (or, rather, with there actually being a breach) here follows a more clearly illustrative example: Think of an organization that has a master administrator, who has control over the whole information system. Imagine that someone takes her children hostage. Now, according to the CIA definition security is not breached until the hostage-takers force the administrator to do things that affects the confidentiality, integrity, or availability of the system. However, even if the hostage-taker is stopped before she does that, the security of the system was clearly breached (for a while).^23^ All other conclusions seem clearly counterintuitive, since before the hostage-taker was stopped she had (indirect) absolute control of the system and the case that an adverse party (the hostage-taker) could do whatever she wanted with the system (during a certain period of time) is reason enough to consider it insecure (during that period of time).

Before ending this section, another aspect of these scenarios is worth mentioning. It is reasonable to argue that Snowden’s reasons for his actions were caused by an ethical conflict with NSA. According to Snowden himself, he did report “to more than ten distinct officials” what he thought was problematic; “none of whom took any action”, and as far as the authors can tell this has not been disputed.^24^ For analysing such ethical aspects of information security, however, the CIA definition offers no help. Indeed, it is perfectly blind to such a security breach. While the CIA definition is compatible with the recognition that a soft issue, such as an ethical conflict, may *lead* to a security breach, it is not, however, compatible with the idea that the soft issue, in itself, can *constitute* the breach (since according to the CIA definition only the violation, i.e. non-retaining, of the CIA properties can constitute a breach of security). The Snowden/NSA example illustrates that a security breach can be constituted by other properties. Again, as was argued earlier, it was the breach that *led* to the violation of the CIA properties, not the other way around. This is part of the reason why the ability to properly analyse security breaches is such a vital feature for a proper definition of the security of information.

So the problem with the CIA definition is not only its inability to deal with contextual variations of the needs of particular organizations, it is also that it has incorrectly conflated security breaches with the consequences of security breaches, and that it is inapplicable to situations that are insecure because of various soft issues, such as ethical conflicts. In sum, there are both actual and possible counterexamples to the CIA definition, and there are counterexamples which show that the CIA definition is analytically false (i.e. it has the wrong sense), and there are examples which show that it fails to be helpful in a proper analysis of security breaches.

## The Appropriate Access Definition

In this section the authors will present and argue for a new definition of information security, the appropriate access definition. This definition will be argued for in relation to some of the fundamental problem previously raised against the CIA definition. Consider the last discussion in the previous section, in which it was argued that the analysis of the Snowden-NSA incident showed that the security breach was partly constituted by an ethical conflict between Snowden and NSA. Taking another perspective on the issue one may conclude that Snowden acted in conflict with what NSA needed, and wanted, in order to achieve security (given NSA’s perspective). As such, although he was authorized, he should not have been so. This fundamental relation—between the needs of a stakeholder and those able to affect its security—is, if spelled out properly, the basis of information security. The general definition scheme, the Appropriate Access definition (‘AA’), thus describes a relation between an object of security, an agent,^25^ and a stakeholder:

*AA (general)*: The object O is secure for stakeholder H if, and only if: For every agent A, and every part P of O, A has just the appropriate access to P relative to H.

This scheme may be applied either to information, or to an information system^26^:

*AA (information)*: The information I is secure for stakeholder H if, and only if: For every agent A, and every part P of I, A has just the appropriate access to P relative to H.^27^

*AA (information system)*: An information system S is secure for stakeholder H if, and only if: For every agent A, and every part P of S, A has just the appropriate access to P relative to H.^28^

Given the main aim of this article, the above definitions specify absolute security, i.e., the state of being secure. However, they can easily be modified to suit a more gradable sense of security.^29^ Henceforth, although the focus will be on this absolute sense of security, it should be clear, *mutatis mutandis*, how the upcoming examples would apply also to some such gradable conception of security.

Before proceeding to further motivate this definition, the basic question of how the term ‘appropriate’ should be understood needs to be addressed. The answer to this is directly correlated to why the AA definition treats security as a property that is relative to a certain stakeholder. According to the AA definition some information (system) may be secure relative to one stakeholder but not relative to another. In other words, on this option, security is relativized to a particular stakeholder. The alternative would be that some information (system) is secure regardless of stakeholder. The two options may be compared with a case of rainfall. The case that it rains is not stakeholder-relative. Whether it is *good* that it rains, however, is plausibly a stakeholder-relative fact: for the farmer, it may be good, although not for the tourist.

We suggest that the most plausible interpretation in the context of information security is the first, stakeholder-relative interpretation. A database system used by an organisation to inform the public, and hence grant everyone access to its files, might be totally secure given the needs of that organisation, while that system is surely *not* secure relative to the needs of another organisation that has another stake in the information, which requires that it is more strictly handled.^30^ In what follows, security will primarily be discussed relative to some stakeholder. On this reading, the question, strictly speaking, is thus not ‘Is the information secure?’, but ‘Is the information secure *for stakeholder H*?’. This feature of the definition enables it to be used to explicitly to clarify conflicts due to different stakeholder-perspectives. The upshot of this suggestion is that ‘appropriate’ should be given an instrumental, ‘appropriate- for’ or ‘relative to’, i.e. the appropriate access is the access that is appropriate, or necessary, for the stakeholder from whose perspective the security property is evaluated.

The formal characterizations of the AA-definitions may be rephrased to enable us to more easily capture the problem in the current situation.^31^ For example, one may speak of an agent not being the appropriate kind of agent^32^ (given the access relations she has), which of course would be equivalent to the agent not having the appropriate access relations (given who she is and how she behaves).^33^ Now, given that security is to be assessed in relation to a stakeholder, for each application of the definition one must operationalize the property of *just the appropriate access* in relation to the needs of some stakeholder (e.g., a particular organization, or individual).^34^ As such the AA definition manages to deal with the time-lock, and other related counter-examples, because if security is retained when information is time-locked it is because this is in line with the stakeholder’s needs (i.e. it is part of what is *just the appropriate access* relative to that stakeholder). Hence the definition achieves stability over time. It allows both for historical analysis, for which the CIA definition may be highly unsuitable,^35^ and it will be compatible with any future developments. It is highly unlikely that one can foresee all the possible needs and threats of the future, but because of the contextual sensitivity of the AA definition it will be perfectly applicable to all such cases. This is particularly important since the field of information security has gone through several paradigmatic changes in fairly short time, transitioning in only a few decades from security as a mostly technical problem (with mainframes) to security as a management problem, and thereafter to yet new paradigms.^36^

Furthermore, one can explain the security breaches in scenarios S1–S3 by referring to the fact that an authorized user at some moment ceased to be the appropriate kind of agent (or equivalently that the access he had was no longer *appropriate*). Because c-Snowden was no longer the appropriate kind of agent for NSA, he should no longer have that kind of access to the information that he had. Lastly, here follows a sketch of how the AA definition is also fully compatible with a gradable conception of security. Firstly, the quantification of the degree of a breach is relative to the importance of those access-relations which aren’t *just appropriate*. Secondly, if one is more interested in the likelihood that some information (system) is secure one need only to quantify the likelihood that the access relations are just appropriate.^37^ Such quantifications are worthy of further discussion, but as previously stated it will not be the focus of this paper. This issue will thus be ignored henceforth.

A good way of pointing to the appeal of the AA definition is to treat what may be the most natural objections from the CIA camp. Thus, before turning to additional (pro)arguments for the AA definition, three objections to the AA definition will be discussed.

*Objection: the AA definition is too vacuous to be useful*. The impression that the AA definition is vacuous or non-informative is natural, in the light that unlike the dominant CIA definition, the AA definition does not specify any substantive features which cause the information security to be retained. But this aspect of the AA definition directly reflects the authors conviction that it is the substantive feature specification of CIA that makes it too broad, and too narrow, as well as analytically incorrect. The CIA definition fails because it attempts to provide a definition as well as a checklist on security features, and as the counter-examples have shown this is erroneous because the checklist would need to vary with the context. Conversely, the AA definition is exactly as *open*-*ended* as it needs to be to properly demarcate the contextually sensitive nature of security. The case is comparable with definitions of moral rightness. Attempts to define the concept in substantive terms have without exception failed, with only comparably non-substantive contenders such as ‘the act we desire to desire’ or ‘the act having the best consequences’ gaining partial support by moral theorists.^38^ With some concepts, there is just too little descriptive content to find a substantive definition which supplies necessary and sufficient conditions for a concept. Even if the morally right act also is the kind act, sometimes refraining from kindness and instead be just constitutes the morally right thing to do. And as the arguments above have attempted to show, the analogue case goes for the CIA properties, as well as other substantive features that often, but not always, are conducive for security. While security has more descriptive content than the paradigmatically ‘thin’ concept of moral rightness—in the AA definition explicated by the appropriate access to an object (information)—the argument is that attempting to add more substantive properties to the AA definition will invalidate it, making it susceptible to the same kind of counter-examples as that used against the CIA definition in the previous section.

Indeed, the seemingly vacuous nature of the AA definition is what enables it to fit with the varying needs of different stakeholders. This rests on the previous analysis of the failures the CIA definition. Part of the problem for the CIA definition was the attempt to claim that all systems require retaining the same properties. Thus, the suggestion is that a correct definition of information security needs to be just as ‘non-substantive’ as the AA definition in order to avoid suffering from counter-examples.

Returning to the argument against the usefulness of the AA definition it is important to point out that while AA is a definition and not, in itself, a method for enhancing security, it can still be of help in the security analysis. In order to be useful not only for grasping the concept but for enhancing the security of an organisation, one needs to operationalize the definition. That is always the case, but due to its explicitly open-ended nature—which is needed, in order to get the correct demarcation of security—this is clear from the start when it comes to the AA definition. The AA definition states that information security is a matter of giving the appropriate access to every piece of (an) information (system). In order to understand what that means in relation to some particular organization, one *need* to analyse the system in context; an analysis that needs to be adapted to the specific stakeholder’s purposes for some information (system). In performing such an analysis it can be helpful to not only think in terms of which access-relations that are appropriate, but equivalently to think about who is the appropriate (or inappropriate) agent for specific purposes (both for humans and technical entities), what just the appropriate access entails for these agents, and what the relevant parts of the information (or system) is, to which these agents should have the appropriate access.^39^ Also, by considering security in terms of the AA definition this enables the unification of both the technical side of information security and the softer side of security under one definition, because appropriate access is operationalized relative to all relevant aspects (i.e. both technical and soft issues). It is instructive to compare the flexibility and analytically open-ended nature of the AA definition with the CIA definition in this respect. Since most people in the field are already familiar with the CIA triad, one may get the impression that it is sufficiently operationalized and ready to go. On proper investigation, however, is clearly not the case. How should we, for example, assess the balance between the central properties *confidentiality*, *integrity* and *availability*? The problem here is not that the actual CIA definitions are varying,^40^ but that regardless, it needs to be modified in practice. As previously discussed (and recognized by professionals), the individual CIA goals potentially conflict with each other, e.g., a too large focus on confidentiality can hamper availability.^41^ In practice this is solved by operationalizing the CIA definition in accordance with the needs of some particular stakeholder. For example, the intelligence services might focus on confidentially, while for many Internet services availability might be more important. But the fact that one can solve these problems does not save the CIA definition, since no CIA definition can include a proper priority between the properties of the CIA triad (because the priorities differ between various systems).^42^

*Objection: giving up CIA is detrimental to current practices*. The second type of criticism relates to the above discussion; many working in the technical field of computer science will probably say that they will never give up the notion of CIA, since it provides a both helpful and familiar tool for analysing information security. But this criticism is in fact off the mark, since what is disputed in this paper is not the importance of the CIA triad. What is disputed is the CIA triad interpreted *as a definition* of the state of secure information. Thus, this does not mean that the AA definition should completely replace the CIA triad when analysing security. The proposal is that the AA definition should replace the CIA *definition*, but that the CIA triad can, and should, still be used under the AA definition, in the sense that they point to important aspects of retaining information security (valid for many systems, even if not for all).

One way of expressing this is that the AA definition provides the *benefit* of being able to utilize the insights that the CIA triad represents, without the problems coming from attempting to use them as a definition of information security. The AA definition is completely compatible with CIA insights, and for all those systems for which it is purposefully applicable, the AA definition can be operationalized to cover this. To illustrate: the concept of *confidentiality* is about who should have access to what—the appropriate ‘who’ to the particular ‘what’ (i.e. just the appropriate access). *Availability* puts requirements on what just the appropriate access ought to be for all or for some specific agents, e.g., timely and reliable (if applicable for the needs of some specific stakeholder), and *integrity* demands that information and system are only accessed in such a way that integrity remains (which for different systems could mean very different things and thus needs to be operationalized accordingly; however, all such operationalisations can be done in terms of what is just the appropriate access). Thus, also from a pro CIA triad perspective, the AA definition is able to harbour all relevant aspects of information security.

*Objection: the CIA definition can be saved by modifying the list of properties*. While this is easily said, it is very hard to picture in actuality. Simply *adding* more properties or aspects—as is sometimes suggested^43^—will not suffice, since, as has already been argued, the CIA definition is both too broad and too narrow. The actual base properties of Confidentiality, Integrity and Availability would have to be modified as well. Here, the proof is in the pudding, naturally, but there is good reason to be sceptical to such a strategy on general grounds. The problem is not a lack of certain properties, but that the definition is neither open-ended enough making it insufficiently ‘flexible’ to properly adjust to contextual difference, nor are the properties of the adequate type for analytical correctness, since it conflates security breaches with the consequences thereof (because the properties are the type of properties that may, for some systems, be reasons for protecting security, rather than being the demarcation of security as such). More importantly: what the previous counter-examples aimed to show (beyond the fact that the CIA properties are neither necessary, sufficient, nor analytically correct) is that there is a contextual normative nature of security that cannot be defined by descriptive properties that hold for any information (system), since this varies with the information, system, and stakeholder. Thus, it is concluded that information security must be defined normatively, as the AA definition does.

One suggestion then would be to complement CIA by normative properties. Dhillon and Backhouse’s principles of RITE (responsibility, integrity, trust and ethicality) have been suggested as addition to CIA in order to address security in organizations, “without which future organizations are doomed”.^44^ But Dhillon and Backhouse seem to suggest RITE given that organisations become less and less hierarchical. Thus, RITE is invented for a specific kind of organisation rather than as properties that can be generally applied. Indeed, while RITE bring forward important principles, they are hardly necessary. Think of an organized crime organization. Their basis for information security is probably based on fear, revenge, and, perhaps, loyalty. Thus, while RITE is hardly applicable to organized crime organizations, the AA definition is applicable to any organization (since it can be relativized to any stakeholder) and can deal with any of the perspectives (further examples of this will be presented in the next section). Thus, the idea of adding properties—intended to cover the soft issues of information security—will end up with the same kind of problems as CIA in itself, for it is highly unlikely that there are substantial and yet necessary properties humans should have that are suitable for the security purposes of any stakeholder.

What the AA definition does by supplying a more open-ended characterisation is to put both soft and hard issues under *one* definition. Using the AA definition, i.e. operationalizing it for your purposes, entails a unified characterisation of secure information rather than, as when attempting to modify the CIA definition, to add two distinctly different kinds of goals, or subsume soft issues under hard issues.

## Further Arguments for the AA Definition

We will now turn to more direct and positive arguments for the AA definition. It has previously argued that there needs to be a proper relation between the analysis of security breaches and the definition of security. Furthermore, it has been shown that the CIA definition lacks such a relation, since it is unable to demarcate between secure and insecure states when these are differentiated by soft issues. A strong feature of the AA definition is that it is not merely a list of properties to retain or goals to achieve, but that it can be used for analytical purposes to understand potential security risks, and then operationalize it accordingly.^45^ It will now be shown how the AA definition may be applied for such purposes, with a particular focus on how to incorporate soft issues. This will be done by returning to the well-known case of Edward Snowden and the NSA. For space reasons, as well as argumentative reasons (to stop potential factual disagreements from getting the points across), the discussion will closely align to the previously discussed idealized version of the story. The discussion will begin with two of the properties of RITE.^46^

*Integrity/ethicality.* The general story of Snowden/NSA is quite well-known, despite the fact that many particulars remain unknown. As described previously: before collecting and leaking hundreds of thousands of documents Snowden reported to some officials the problems with the illegal, highly privacy invasive activities of NSA. Given the available information, the non-action of these officials gives important clues as to his reasons for leaking the information. In the following discussion the aim is not to ethically assess these events in terms of moral rightness and wrongness. The aim is to show that considerations about ethics and values can play a constitutive part in security breaches (indeed, as will be argued, it was the moral convictions of Snowden, in conjunction with his willingness to act, that resulted in, and was constitutive of, the leak).

Now, the simple analysis is that Snowden leaked information because his ideals conflicted with those of NSA.^47^ Snowden thought that what NSA was doing was wrong. A central aspect of the operationalization of the AA definition is the question of what kind of agent that is appropriate for a certain access-relation to a certain piece of information (or, e.g., which access relation that is appropriate for that agent). Accordingly, had NSA analysed the situation using the AA definition in relation to the contextual data they had available, they might have concluded that Snowden was not the appropriate kind of agent (because he was behaving inappropriately given the perspective of NSA). On the other hand, if one applies the perspective of another stakeholder on the issue, e.g., the perspective of many Americans (and others), it seems as if he was *exactly* the appropriate kind of agent: exposing *their* security infringements was relative to these stakeholders the appropriate thing to do. In a nutshell, it was this inherent ethical conflict that led to, and was partly constitutive of, this very famous security breach.^48^

In order to understand this ethical conflict it may be illustrative to consider an example where the perspectives are more clearly diametrical. Take the cold war setting of US and Soviet and consider some secret US information that a spy has acquired, with the goal of selling it to the Soviet Union. Is the information secure? Well, that depends on what perspective one takes. From the US perspective the information is not secure, but would again be if it is retaken and the spy is, e.g., eliminated. On the other hand, from the Soviet perspective the information would be secure if they can retrieve it from the spy. This value-conflict is fairly obvious, but what is interesting about the Snowden/NSA situation was that this value-conflict was not external (as in the US/Soviet case) but it was an internal conflict within the organization. Thus, there was a goal conflict within the organization that motivated an agent (Snowden) to act contrary to what is appropriate for the stakeholder (NSA).

The AA definition can be used to expose security risks due to such value conflicts, because it allows one to reason from various perspectives (relative to different stakeholders) and what would be just the appropriate access according to the perspective of this or that stakeholder. Once one has operationalized the AA definition in accordance with the perspective of a certain stakeholder, e.g., that of NSA,^49^ then one can see if that perspective risks conflicting, e.g., with the moral perspectives of their employees, consultants, or external agents. Such a value-conflict is not necessarily a security breach, which arguably also would require a willingness to act. But any conflict with the operationalization of the AA definition shows a security risk (a threat or a vulnerability—the difference is for this discussion irrelevant), e.g., that people may choose to act on other goals (e.g., efficiency or privacy) if fully motivated to do so (which in the case of Snowden obviously was the case). Thus, one may conclude that the AA definition is useful for exposing security risks and therefore avoid potential security breaches. The actual demarcation hangs on the conflict being substantial enough to be a cause for action. This is in itself a complex philosophical issue, which has to be discussed elsewhere.^50^ However, this brings up the next pro-argument concerning the AA definition’s relation to analysis.

*Exposing false trade*-*offs.* The ethical dilemma shows yet another interesting problem that the AA definition can expose; false trade-offs. Now, consider the fact that Snowden reported what he saw as problematic to some supervisor, and that his reports were ignored. One might think that NSA opted for security over privacy, i.e. that there was a trade-off between the privacy (i.e. the privacy of the American people) and the information security of NSA. Admittedly, it is actually slightly more complicated since the goal of the trade-off is public safety or national security. In this trade-off it is reasonable to argue that information security (mainly in terms of the secrecy of the spy programs) were a means to this end, since by keeping the program secret it is reasonable to think that it takes less of an effort to defend against measures that the adverse parties (e.g., potential terrorists) do not know of. As previously noted, it was this secrecy that Snowden took conflict with (see fn. 47).

On closer analysis, what *seemed* to be a trade-off was not a proper trade-off. When Snowden reported the privacy problems to NSA it is likely that they thought a trade-off between privacy, or a right to consent, and secrecy (i.e. information security of their spy programs) was possible. However, a successful trade-off depends on one value actually being traded for another value—in this case the right of the public to consent to privacy invasive activities for increased security via secrecy. But, while NSA did trade-away the people’s right to consent/privacy it is clear that they did not get any increased information security by secrecy in return. Since their actions caused an information security (secrecy) risk, a risk which was also actualized. Arguably, Snowden leaked hundreds of thousands of documents *because* NSA failed to act on his reports; something he most likely would have refrained from doing, had NSA taken satisfactory measures to protect the privacy (of the American people) and/or given the public a chance to consent. Thus, not all trade-offs are successful. In particular, NSA’s trade-off between privacy and security failed to some extent; specifically, what failed was that part of the trade-off, or that specific trade-off, which focused on increased security through secrecy for a decrease (infringement, or violation) of the people’s privacy and/or their right to consent.

The problem is that when NSA traded privacy (and the public’s right to consent) for security (and security via secrecy), they did not trade-away the ethical conflict, and this ethical conflict was part of Snowden’s reasons for leaking the documents (i.e. it was substantial enough to be a cause for his actions). Consequently, the trade-off between privacy/consent and security via secrecy was a false trade-off that ended up creating a security risk that in fact was actualized. Generally, one needs to realize that actions that are ethically loaded will carry their own security risks.

These are the kind of risks that the AA definition can expose, since if one operationalizes the AA definition in accordance with what presumably would be *just the appropriate access* for NSA, then the appropriate kind of agent is one that submits to such ethicalities (one that shares their priorities). The purpose of this example is not to judge the morality of NSA’s action. However, it is clear that the example concerns an ethical grey zone—or a moral dilemma—which was a problem for their information security. In fact, one can conclude that how they dealt with the value conflicts of the situation was an important part of what caused the security breach.^51^

A general problem with these ethical dilemmas is that employees may, or they may not, be sensitive to them (and there is of course a risk that the latter category may also be insensitive to other ethical or moral issues). There are no general ways to trade off (or trade away) ethically loaded issues without having such effects on security. Since there is no simple way to trade between ethical considerations and security, if one wants to solve the effects on security due to ethical conflicts, then the conflicts as such must instead be solved. A virtue of the AA definition is that such conflicts can be identified by considering the various possible perspectives different agents could have as stakeholders, and analyse if security on those perspectives conflicts too severely with the perspective of the actual stakeholder.

*Analysing different perspectives.* From the discussion above, it can be concluded that if NSA would have operationalized security in terms of the AA definition, and analysed the situation from all relevant perspectives (e.g., by considering Snowden’s perspective), then they would most likely have identified a security breach within NSA, since Snowden was obviously not the appropriate kind of agent for NSA (and thus that he had access he should not have).^52^

Consequently, one of the benefits of the AA definition is that it offers *one* definition that is suitable for *any* perspective, concerning the state of security. Thus, the AA definition is not only a better definition than the CIA definition because it properly demarcates information security, and is sufficiently fundamental; it is also a better starting-point for studying the security from any perspective. This is because the AA definition matches any proper analysis of a security breach. Thus, used properly, the AA definition promises to be helpful not only for security viewed as a technical problem, but also for studies of the soft issues of information security that do not need to use CIA as a comparative measurement.^53^

The AA definition may be utilised as a way to make comparisons between individuals and organization in order to analyse security risks, relevant cultural issues and other central security notions. By comparing conceptualization of the AA definition from different perspectives (including both malicious and non-malicious agents) one can also discover, e.g., weaknesses, ways to improve security, or unnecessary security measures. Since the AA definition allows any perspective to be operationalized, it is a far better starting-point than the CIA definition. Furthermore, if one is interested in studying, e.g., how security perspectives varies in organizations, how such variation relates to overall organizational culture, or how it deviates from the organization’s security goals, the AA definition is a not only a superior definition but of more fundamental use in the analysis of information security.

Lastly, while space reasons have not permitted any further development of such an analysis in this paper, it should be clear from the examples given above that the analysis of, and possible resolution to, the conflicts between privacy and security, or different perspectives on security, can be helped by the AA definition. Thus, its virtues extend well beyond the direct applications discussed here.

## Conclusions

It has been demonstrated that the CIA definition is susceptible to certain counter-examples, which shows that its supposed necessary properties aren’t necessary, that it does not sufficiently recognize that security needs vary with the context, that it incorrectly conflates security breaches with its consequences, and that the CIA definition simply doesn’t match up with the proper analyses of security breaches and incidents. Hopefully, it has been shown that these problems are serious enough to abandon CIA as a definition.

The open-ended nature needed in order to have a definition that is fundamental enough to capture the phenomenon cannot be on the detailed level of the CIA definition. The CIA definition is not flexible enough to deal with every situation and the varied requirements from different information, systems or organizations. It neither does, nor can, include human effects on security in a proper manner into the definition, especially not in a way that analytically identifies what the breach is, or when it occurs; nor does it supply necessary reasons for having security.

The AA definition, on the other hand, captures the notion of security of information both in terms of demarcation and in terms of explanatory salience (relating properly to analysis of security breaches and incidents). Its open-endedness guarantees its temporally stability, since future changes to the underlying properties of a system which enhances, or decreases, security will only affect the operationalisations of the AA definition, not the definition as such, irrespective of how radical such future changes may be. Also, the AA definition is flexible enough to deal with all possible situations, the soft issues, and the varied requirements of different information, systems, or organizations. It is concluded that rather than trying to patch the CIA definition to become a proficient definition, the AA definition should replace the CIA definition as the definition of secure information, while the CIA properties should be perceived instead as a useful (albeit fallible) rule of thumb for identifying important security aspects. The AA definition enables studies of the soft issues of security—including, e.g., humans, organizations, culture, ethics, policies, and law—by being a definition that recognizes all fundamental elements of security.^54^ It thus bridges the divide between the hard (‘technical’) side of security and the soft side security, supplying *one* definition that encompasses all perspectives and is useful for any relevantly related security purpose. Lastly, a further virtue of the AA definition is that it is helpful beyond analysis of pure security issues, such as conflicts between privacy and security.

## Footnotes

1 Throughout the paper, the issue of information security is discussed in terms of *information (system) security*, discussing both the question of whether some information is secure and whether some information system is secure. The intension is not to conflate these issues, rather they are discussed in tandem to simplify the discussion. Part of the reason is that the former often depends on the latter. For example, if I put some information in a vault and hang the key to the vault outside, then the information is, arguably, insecure because the system is insecure. Many of the examples that will be discussed hinge on such cases. Also, since the definition that will be proposed can be applied to both, it is therefore worth to make sure, to the extent it is possible vis-à-vis spatial limitations, to widen the discussion already at an early stage.

2 Gupta (2015).

3 Ibid.

4 In order to simplify, ‘security’, ‘information security’ and variations thereof, will be used as synonymous with ‘the security of information (systems)’.

5 ISO/IEC 27000 (2016: 3, 4, and 7). Italics (indicating a definition of a term) removed from ‘processes’. Henceforth the ISO-standard will be referred to as ‘ISO’.

6 Ibid: 6 lists four properties that can be added: authenticity, accountability, non-repudiation, and reliability (all of which are further defined).

7 See, e.g., ibid.; United States Code, Title 44, Chapter 35, Subchapter III, § 3542—Definitions; NIST Interagency Report (IR) 7298 Revision 2 (2013). *Glossary of key information security terms* (which includes several references to other national US standards that apply the U.S.C. definition to some extent: SP 800-59, CNSSI-4009, SP 800-37, SP 800-53, SP 800-53A, SP 800-18, SP 800-60, FIPS 200, FIPS 199, SP 800-66), Bishop (2005), Peltier (2001), and Nissenbaum (2005).

8 There are various opinions of the relation between the CIA triad and information security, some view the CIA triad, or an extended version of it (i.e. including other security terms), as “the foundation of information security” (Tiller and Fish 2004: 223), as something information security “rests on” (Bishop 2005: 1), as categories “[t]hreats generally fall into” (Chapman 2004: 342). Others have treated CIA as something that superficial might seem closer to providing goals; e.g., direct goals as in “computer (and network) security has been defined by three goals” (Nissenbaum 2005: 63), or more indirectly, i.e. information security is supposed to “provide” CIA (44 U.S.C., Sec. 3542), “preserve” it (ISO: 6; cf. Peltier 2001: 4; Parker 1998: 230, 2009, “protect” it (cf. Takanen et al. 2004: 93), yet others view it as “the sum of its component parts” (Solomon and Chapple 2005: 2), that discussions about it “mean that we are addressing three important aspects of any computer-related system” (Pfleeger and Pfleeger 2007: 10) and yet others view it as “basic security requirements” (Pieprzyk et al. 2003: 3).

9 Indeed, it is well known in the literature that the CIA properties can even conflict with each other (cf., e.g., Pfleeger and Pfleeger 2007: 10).

10 Since there is, as has been noted (cf. fn. 8) vast disagreement on the role the CIA triad in a definition the main task of this paper in relation to the CIA triad is to test whether the CIA properties can serve as necessary and sufficient conditions to define the demarcation between secure and insecure information (systems).

11 One of the reasons that this is most fundamental is that there are, as was previously noted, various ways to conceptualize a gradable conception of security, e.g., in terms of the severity of a breach or the likelihood of breach.

12 See, e.g., Dhillon and Backhouse (2000), and Dhillon (2001a: 165, b: 2).

13 This is well known in the literature (see, e.g., von Solms 2001a). Thus, the aim is not to present this as a new insight, but to show that the CIA definition is ill-suited to serve as a definition of information security *given* such insights.

14 ISO: 3.

15 See Pfleeger and Pfleeger (2007: 10) for a discussion of the need for prioritisation of the CIA properties.

16 That said, the authors believe that for most sensible systems integrity will, for at least some part of such systems, be a central property. Thus, the lion part of all systems/information need integrity to some extent, but since it is not necessary for all information (systems) it cannot be part of the criteria of a definition. Furthermore, it is reasonable to think that even for the information/systems which require integrity (to some extent), what they specifically require varies from system/information to system/information. Part of the problem is, thus, how to construct a definition that can make sense of the virtues of integrity (as well as confidentiality and availability) in a way that resolves the weakness of the CIA definition. It will be discussed how to map these properties to the Appropriate Access definition, once it is presented, in “The Appropriate Access definition” section.

17 Thanks to an anonymous referee for pointing this out.

18 As will become clear in the main text below, this ‘should’ should not be taken as indicative of an ethical ‘ought’. Rather ‘should’ here operates over the purpose of the information, system, and/or organization.

19 See “Sweden Sinks Into Political Chaos After Classified Data Breach” (Niclas Roalander, Anna Molin, and Hanna Hoikkala, Bloomberg Politics, July 27, 2017, 8:43 a.m. GMT+2 (http://www.bloomberg.com/news/articles/2017-07-26/swedish-government-faces-no-confidence-votes-after-it-scandal).

20 This is mentioned as plausible (“it seems that”) in *The Economist* (http://www.economist.com/news/international/21580191-edward-snowdens-odyssey-leaves-america-nonplussed-and-its-allies-dismayed-russia-china).

21 The idea of c-Snowden is that he retains the familiar properties of Snowden as someone who gathers information in order to disseminate it (i.e. a whistleblower). However, contrary to the actual situation, c-Snowden is authorized to access all the information he gathers. It is important to note that there is nothing erroneous, per se, from a security perspective, to gather information (unless, of course, by doing so you also risk exposing the information). For example, the NSA (National Security Agency) most likely attempted to gather exactly the information Snowden leaked in order to evaluate the effects of the leak. Thus, the purpose of revising the example of Snowden is that c-Snowden allows one to analyze the demarcation between a state when the gathered information is secure and a state when it is not secure.

22 Of course, one may reason that the likelihood of further dissemination is larger in S2 and S1, but one could imagine a situation similar to the above scenarios, in which T is the *only* one not authorized to access the information. According to a gradable version of the CIA definition the information would then be less secure in S3 than in S2 and S1, since T did access the information. But if T is dead and did not have a chance to do anything with the information (e.g., affect its integrity) it seems that the information is secure although the confidentiality criterion has been violated. Again, the only way to save the CIA definition would be focus on the value of the consequences (arguing that S3 is *worse* than S2 and S1, because it mattered whether T accessed the information, not what he could do with it). However, while this may be possibly true for some obscure cases it does not hold with generality.

23 If one focuses on information systems security, rather than information security, and if one considers the administrator to be part of the information system, then security is breached according to the CIA definition already when her children are taken hostage (because it can be argued that it affects her—and therefore the system’s—integrity). However, this way of reasoning is self-defeating since on such an analysis the administrator taking a day of sick-leave would affect integrity and therefore security, which obviously does not follow with any necessity.

24 See Snowden’s testimony to the European Parliament (http://www.europarl.europa.eu/document/activities/cont/201403/20140307ATT80674/20140307ATT80674EN.pdf).

25 The term ‘agent’ refers to any entity (technical, software, human etc.) that may have some kind of access to some part of the object.

26 In this paper the authors will remain partly neutral as to the demarcation of an ‘information system’ (see, e.g., fn. 23).

27 The AA definition—just as the CIA definition—is a definition that is expressed relative to the concept of information. There is a broad debate on the concept of information (see Adriaans 2013 for an overview). However, the authors believe that the AA definition is orthogonal to the debate on the concept of information. What will change by applying a different kind of definition of information is the security-object (i.e. the step from O to I). Now, certainly the AA definition will seem less convincing relative to some theories, but that just says something about the limits of those theories in this context. If a definition of information is too narrow, then the problem is not the AA definition as such, but that the operationalization of the security object is too narrow (a concept which is not attempted to define in this article). For example, if one has a very narrow definition of information which states that only the content of the US constitution is information, then it may seem strange that security of information should apply only to this object—but this claim does not follow from the AA definition, but from the definition of information. Thus, it is the definition of information that is in need of modification, not the AA definition. It is also worth to note that if the AA definition is applied to such a definition of information, the AA definition would then define when such an object is secure. Broadness, on the other hand, hardly seems problematic at all, since it is hard to imagine a definition of information that causes any (security) problems because the definition of information is too inclusive. Take, for example, a broad proposal such that information is well-formed data. A potential problem would then be that there would be information (according to the definition) that does not need any security. However, given the AA definition that is not a problem, since that would just mean that any access to that information is appropriate. The only problem with a broad definition is that it may be too inclusive in the sense that the security issues are not only about information, but about security in general. Again that is an issue that does not require any alteration of the proposed definition, but of the background theories of information as such.

28 In the main text, information will be treated as a part of an information system. However, if one wants to separate them, a combined, slightly more complex definition is easy to construct: An information system S and some information I is secure *iff* for every agent A and every part P_1_ of S and P_2_ of I, A has just the appropriate sort of access to P_1_ and P_2_.

29 If one wishes to define a gradable conception of security in terms of the degree to which some information (system) is secure, then this can be achieved by replacing ‘if, and only if: For’ with ‘to the degree that’. If one is more interested in quantifying the severity of a security breach, then this has to be evaluated by quantifying the importance of each kind of access.

30 This does not imply that the authors deny that one possibly can make sense of information security as a stakeholder-independent property in the above sense. For example, it may be argued that a security statement, ‘Information system S is secure’ is essentially context-dependent, and so should be assessed given the actual context in which the statement is uttered. So when the CEO of the organisation utters ‘our computer system is secure’, it is just as little an objection to that claim to retort ‘not from my perspective’ than it would be to object to someone claiming ‘I am right-handed’ by saying ‘No, I am left-handed’. While on a weak interpretation, this option is essentially another way of saying that the stakeholder-perspective is implicit in the claim, a stronger interpretation would be to include a claim that some perspective is privileged in a stronger sense. The rivalling organisation in the database case above, for example, might successfully argue that since the information should be kept secret, or at least not available to all, the information is in fact not secure, even if the system functions as intended by the system owner (who wants the information to be public). This option will not be pursued further, however, since the perspective-relative interpretation in the main text suffices for the intended purposes.

31 It may be illustrative to think of the goal of security as ‘appropriate agent, appropriate access, and relevant information’, i.e. the appropriate agents ought to have the appropriate access to the relevant information.

32 The concept of appropriate agent is a complex depending on two properties: being a person whom under the right condition should have access and being a person that behaves appropriately (given the access she has). See fn. 39, for an illustrative example of the conflicts this can lead to.

33 Typically, few persons are, or act, so inappropriately that they should have no access to an information system. One may want—or at least allow—even the most notorious hacker to be able to access one’s website. However, this does not imply that one wants her to be able to perform, e.g., a DOS-attack.

34 One might notice that the AA definition fits well with certain policy approaches. Pfleeger and Pfleeger argues that: “A security policy must answer three questions: *who* can access *which* resources in *what* manner?” (see Pfleeger and Pfleeger 2007: 547).

35 Given, e.g., that the use of main frames had other general security problems.

36 See von Solms (2006), cf. also von Solms (2001b).

37 Cf. fn. 29, for a similar example of how to modify the definitions. Note that the example in the previous fn. defines the degree to which some information (system) is secure. Degree can be read both to include the likehood and a valuation of its importance (as the above example of the valuation of a breach illustrates).

38 See, e.g., Moore (1903), Gibbard (2003), Horgan and Timmons (1990–1991).

39 It is important to note that by speaking of appropriate and inappropriate access (or appropriate and inappropriate agents), it does *not* imply that any access is *either* appropriate *or* inappropriate, regardless of perspective. Furthermore, there are, of course, security-related dilemmas, in which someone’s access is both appropriate (in one sense) and inappropriate (in another sense). Take for example the story that US intelligence agencies have started to withhold information from The White House, because of distrust (see, e.g., John R. Schindler, *The Observer*, February 12, 2017 10:00 a.m., http://observer.com/2017/02/donald-trump-administration-mike-flynn-russian-embassy/). While the authors remain agnostic as to the truth of this claim, the story exemplifies the point the authors wish to make. An even better example: if the President himself cannot be trusted to keep secrets, then there is truly a security-related dilemma, since the President (in one sense) ought to have the information, but (in another sense) is not the appropriate kind of agent (given, e.g., that the American people is the stakeholders). In such cases, AA correctly defines such systems as insecure, because the access is (in some sense) inappropriate.

40 See fn. 9.

41 See, e.g., fn. 15.

42 One may worry that according to the AA definition one cannot determine whether something is secure or not, because, in actuality, one always lack perfect knowledge of what the appropriate access-relation should be. However, this is not a problem, since it is doubtful—except in rare or limited cases—whether one ever truly *can* determine if something is secure. This goes well beyond problems such as knowing who has access to what (something that for various systems can be verified), since as has been argued there is *always* some uncertainty whether people with access ought to have access. The AA definition captures this feature of uncertainty that is an essential part of the practical struggle for security.

43 Cf. Parker (1998, 2009).

44 See Dhillon and Backhouse (2000: 127).

45 The point is not that the AA definition will resolve all security issues. The point is that the AA definition (in comparison to the CIA definition) is compatible with—as well as helpful in the performance of—various kinds of security analyses.

46 Responsibility is already covered in the sense that the three constituent parts of the AA definition may be assigned responsibility, i.e. agent, access, and information—and one may ask who is responsible for each of these being appropriate and relevant.

47 This is in fact supported by Snowden’s own claims. In an interview by James Risen, Snowden argues that it was the secrecy concerning NSAs spy program that he found most problematic, arguing that such programs could have a level of legitimacy if “there’s broad support amongst a people” (requiring, of course, that they are—to some extent—informed). See “Snowden Says He Took No Secret Files to Russia”. *New York Times* October 17, 2013. http://www.nytimes.com/2013/10/18/world/snowden-says-he-took-no-secret-files-to-russia.html.

48 The general importance of ethics is quite well-known. Cf. Dhillon and Backhouse (2000), Trompeter and Eloff (2001), and von Solms (2001a).

49 Now, of course, the authors has limited knowledge of how they would (and should) *actually* operationalize it.

50 The question of causality is a complex one, discussed in many different areas of philosophy, possibly to the greatest extent in philosophy of science (but also in, e.g., meta-ethics when it comes to questions such as free will and moral responsibility). For the purpose of this paper, a commonsensical, non-technical understanding of the notion is sufficient.

51 It is not unreasonable to suggest that this conflict supplies an INUS-condition for the security breach. But as previously noted, cf. fn. 50, these issues will merely be discussed from a commonsensical perspective.

52 One should not mistake the example for intending to communicate that NSA would have avoided this breach by spending more time spying on their employees (or consultants), since spying on your employees would probably lead to even more ethical conflicts and potentially end up in a much greater security breach (as the fall of East Germany may show).

53 On the potential limits of using CIA to analyse, e.g., security needs, cf. Dhillon and Torkzadeh (2006), and Kolkowska et al. (2012).

54 Cf. von Solms (2001a).
